# Lord Mayor Whitehead and the Working Classes

**Published:** 1889-05-11

**Authors:** 


### The Hospital

A Weekly Institutional Journal of

### Science, Medicine, Nursing, and Philanthropy.

*For Hospitals, Asylums, Medical Practitioners, Students, Nurses, Families, Parishes, Congregations, and the Charitable Public.*

Vol. VI. No. 137.] [May 11, 1889.

### Lord Mayor Whitehead and the Working Classes.

Lord Mayor Whitehead is by no means a commonplace man. The set of his face, the attitude of his body, the firm tones of his voice all denote decision, will, and originality of character. In his public capacity the Lord Mayor has very definitely taken up the cause of hospitals. During the last few weeks he has given the lead in a direction where thousands have shown themselves ready and willing to follow. The great meetings held in the four quarters of tho metropolis on behalf of the "Penny-a-Week" Fund have strikingly demonstrated the intensity of the feelings of working men in favour of the institutions to which they owe so much.

The present is undoubtedly a time in which medical charities deeply exercise the public mind. For one reason or another many different classes think much and speak warmly on the subject. Enthusiastic friends who can see no faults in hospitals, unreasoning enemies who can see no virtues, candid critics who have high ideals of perfect management, injured medical practitioners who groan beneath the burden of an oppressive competition, and many others have contributed their stones to the cairn. It is, therefore, a peculiarly favourable time for all the sober-minded supporters of these charities to take up the consideration of the question of charitable medical relief *de novo*, and to make an effort to adapt their methods to the economic and scientific requirements of modern times. To go no farther than the speech which Lord Mayor Whitehead delivered to his enthusiastic audience at Holloway, it is plain from that speech that his lordship has but the most elementary knowledge of the great hospital question. There is no blame to him for that. No man can qualify himself for the post of Chancellor of the Exchequer in a week; neither can even a Lord Mayor become an authority on hospital administration by the studies of an afternoon. His lordship, for example, pointed to the well-known fact that of the 9,480 hospital beds existing in the metropolis, no fewer than 2,637 were unoccupied, and regarded it as a calamity so evident that no one could fail for a moment to see and deplore it. It may be a calamity, or it may not. But this, at least, is certain—that to make an appeal for additional funds for hospitals merely to fill their unoccupied beds is not a method of proceeding which commends itself to practical wisdom. What really should be the aim of sober philanthropists is that every person who needs a hospital bed, and who is entitled by his necessities to claim it, shall not have to wait a day before his need is met. It is quite possible that the 6,843 patients who actually occupy the beds of the London hospitals at the present time, represent all those who have any *bona fide* claim upon that kind of charitable medical relief. But whether that be so or not, there is nothing in the Lord Mayor's speech to show that any widespread suffering is experienced in London on account of the vacant beds at the hospitals. Those who ask for enlarged resources must not be content to show that hospitals could do more charitable work if they had more money, but they must prove that more charitable work imperatively needs to be done.

When the Lord Mayor emphasised the fact that it was the wage-earning class who benefited most largely by the hospital system, and drew from it the conclusion that therefore that class ought to be in the front rank of hospital supporters, he was on far more solid ground. The wage-earners occupy a much better position, both socially and materially, to-day in England than they have ever done at any previous time. The amount of support they have hitherto given to hospitals has been by no means large, and has certainly not been such as to excite the admiration of other classes. They are now at length bestirring themselves, and it will be well for them if they show something like an adequate appreciation of the position. As matters stand at present, the country is face to face with the fact that nearly half its population are quite willing when sick to depend for medical relief upon the charity of outsiders. However that spectacle may strike others, to the present writer it has the appearance both of a national calamity and a national disgrace. If the wage-earners be really so poor that they cannot at all pay for their own medical relief, it is a disgrace to the rest of the commnituy; but if, as we believe, they are so prosperous that at least the majority of them could pay quite well, then it is a disgrace to the workers themselves. No man, whatever his position, ought to accept a single penny from any charitable source until he is compelled by a necessity which is inexorable. The sight of strong men in full work, drinking beer and smoking tobacco when well, and then flying to the hospital at the first sign of illness for the aid of charity, is, if we would but think of it, both a scandalous and a dangerous sign of the times. A professional man is ashamed to depend upon charity; why should not a workman be similarly ashamed? The workman claims to be a man just as much as the other; he demands and receives various recognitions of his manhood, such as the suffrage and the like, at the hands of the Legislature. On what ground, then, can he justify to himself that pitiable lapse from self-respecting decency which is the fate of every man who once accepts unneeded charitable aid?

The Lord Mayor calculated that to occupy permanently the 2,637 vacant beds would necessitate an increased expenditure of a little more than £100,000 per annum; and that sum he considered the enormous working population of London could very easily raise by their penny a week scheme. He mentioned several towns in which larger relative amounts than that had been raised by similar methods. Among others he instanced the case of Sunderland, which, he said, had contributed in workmen's subscriptions between three and four thousand pounds last year. He might have made a very striking point if he had reminded his audience that that was probably more than a fourth of the total hospital expenditure of the town for all purposes. If the workmen of London succeed in raising their £100,000, as they undoubtedly ought, the proportion it will bear to the total expenditure of the metropolitan hospitals will be nearer an eighth than a fourth. Whence comes this discrepancy between the hospital contributions of the Sunderland and the metropolitan working men, and the still greater discrepancy between the hospital supply and expenditure of the two places? That is a question which should engage the most serious attention of all metropolitan hospital managers.

The scheme supported by the Lord Mayor for the raising of £100,000 annually by means of the Penny-a-Week Fund is both large and good. But it is neither large enough nor good enough to confer honour upon the working men who are called upon to support it. Many of them are already singing pæans of self-glorification on the mere possibility of making the scheme a success. Such easily-pleased people have no adequate sense of the real demands of the situation.

The ideal to be aimed at is not the raising of a hundred thousand a year and the appointment of a hundred working-man governors to the various metropolitan charities. It is the complete support of all the medical institutions which treat the prosperous working classes by the money of those classes themselves, the complete support and the complete government. That is an ideal worthy of men. To continue the present system is to continue a system of dependence which is often little better than a degrading slavery. To aim at that degree of self-help which makes for complete independence is to strive for such a measure of emancipation as alone deserves the consideration of self-respecting and far-seeing Englishmen. Of course, Rome was not built in a day, and it cannot be expected that the working classes of London, after being nursed on charity for so many years, will all at once start off on the bold path of complete self-help. Many of them, it is to be feared, have not manliness of character enough even to wish to walk alone. But impracticable as our suggestions may appear at the moment to those who have not studied the question, they raise the only true standard which ought to be set before a progressive people. As a commencement of better things, as the initial step towards the best things, we hail with great satisfaction the Lord Mayor's championship of the principle of self-help for all.

